# The effect of bed rest, unilateral limb immobilization and head‐down tilt on muscle protein synthesis: A systematic review and meta‐analysis

**DOI:** 10.1113/EP092474

**Published:** 2025-10-30

**Authors:** Konstantinos Prokopidis, Paul T. Morgan, Colleen S. Deane, Oliver C. Witard, David D. Church

**Affiliations:** ^1^ Department of Musculoskeletal and Ageing Science, Institute of Life Course and Medical Sciences University of Liverpool Liverpool UK; ^2^ Department of Sport and Exercise Sciences, Institute of Sport Manchester Metropolitan University Manchester UK; ^3^ Human Development & Health, Faculty of Medicine University of Southampton, Southampton General Hospital Southampton UK; ^4^ Centre for Human & Applied Physiological Sciences, Faculty of Life Sciences and Medicine King's College London London UK; ^5^ Department of Geriatrics, Donald W. Reynolds Institute on Aging, Center for Translational Research in Aging and Longevity University of Arkansas for Medical Sciences Little Rock Arkansas USA

**Keywords:** fractional synthetic rate, muscle disuse, physical inactivity, skeletal muscle

## Abstract

Muscle disuse leads to muscle atrophy and a decrease in muscle function that is primarily driven by reduced muscle protein synthesis (MPS). The aim of this systematic review and meta‐analysis was to examine the effect of different models of muscle disuse on rates of MPS. A literature search was conducted in PubMed, Web of Science, Scopus and Cochrane Library. Eligible randomized and non‐randomized controlled trials compared the effect of bed rest (BR), unilateral lower limb immobilization (ULLI), and 6° head‐down tilt (HDT) on pre–post muscle disuse changes in MPS in adults. Using the random and fixed effects inverse‐variance model, we calculated the mean difference (MD) in the effect of BR on mixed MPS and ULLI on myofibrillar MPS (MyoPS) (both expressed as fractional synthetic rate,%/h). The effect of HDT on MPS was examined through a narrative synthesis of data. A total of 16 studies were included in the systematic review and 13 in the meta‐analysis. A significant reduction in mixed MPS was observed after BR (*k* = 4; MD: −0.017%/h, 95% CI: −0.023 to −0.011, *I* ^2^ = 24%, *P* < 0.01) and a significant reduction in MyoPS was observed after ULLI (*k* = 9; MD: −0.015%/h, 95% CI: −0.021 to −0.008, *I* ^2^ = 94%, *P* < 0.01). HDT led to reductions in both mixed MPS and MyoPS. A comparable reduction in mixed MPS and MyoPS was observed between different models of muscle disuse.

## Abstract (highlights)

- **What is the central question of this study?**
  What is the impact of different muscle disuse models on muscle protein synthesis?
- **What is the main finding and its importance?**
  Muscle disuse, induced by bed rest, unilateral lower limb immobilization and 6° head‐down tilt, significantly reduces muscle protein synthesis (MPS). The findings indicate comparable declines in MPS across different disuse models, highlighting their impact on muscle atrophy and function.

## 1 INTRODUCTION

Physical inactivity poses a significant public health issue in modern society. It results from increasingly sedentary lifestyles, a higher prevalence of chronic disease, and/or acute injuries that may lead to surgery (Booth et al., 2012). Periods of disuse and almost complete inactivity (including bed rest [BR] and immobilization) that are typical during illness and hospitalization also result in rapid muscle atrophy, declines in muscle strength and function, and impaired postprandial rates of muscle protein synthesis (MPS) (Deane et al., 2024). In the short term, physical inactivity accelerates the decline in muscle mass and decreases recovery time from injury (Welch et al., 2018), whereas longer‐term consequences encompass an increased risk of falls, hospitalization (and a greater risk of readmission), lower quality of life and a reduction in life expectancy (McKendry et al., 2024).

The detrimental effects of muscle disuse on muscle mass, function and protein metabolism occur rapidly. For instance, 1 week of BR elicited a 1.4 ± 0.2 kg decline in lean tissue mass and a 3.2 ± 0.9% decline in quadriceps cross‐sectional area in healthy, young males (Dirks et al., 2016b). In addition, Arentson‐Lartz et al. (2016) demonstrated that 14 days of BR led to a reduction in vastus lateralis satellite cell content, capillary density and myosin heavy chain type IIa fibre size and aerobic capacity in middle‐aged adults (Arentson‐Lantz et al., 2016). Impairments in mitochondrial energetics have also been observed following 10 days of BR in older adults (Standley et al., 2020). These findings were substantiated by a meta‐analysis that reported that 14–28 days of muscle disuse elicited a marked degree of lower limb muscle atrophy in healthy participants, the magnitude of which was exacerbated in intensive care patients (Hardy et al., 2022). Moreover, reductions in muscle strength have been observed following experimental BR in young and older adults within a 5‐ to 14‐day period (Di Girolamo et al., 2021).

An imbalance between MPS and muscle protein breakdown (MPB) underpins *simple* disuse‐induced atrophy (i.e., not confounded by an underlying disease) (Deane et al., 2024). For instance, 14 days of 6° head‐down tilt (HDT) have been shown to reduce MyoPS in young adults (Ferrando et al., 1997), and similar impairments in MPS were observed after 2 days of unilateral lower limb immobilization (ULLI), with effects persisting for ≥1 week (Kilroe et al., 2020). During periods of physical inactivity, the decline in muscle mass appears to be primarily mediated by a reduction in mixed MPS and myofibrillar MPS (MyoPS), considering that previous research has demonstrated no impact of immobilization on postabsorptive MPB rates (Brook et al., 2022; Pavis et al., 2023). However, since BR rest and HDT represent whole‐body models of muscle disuse, whereas ULLI isolates a specific limb, the magnitude of disuse‐induced muscle atrophy via perturbations in MPS may be considered mode dependent (Dirks et al., 2016a).

To our knowledge, no systematic analyses have previously been conducted to examine the effect of different disuse models on the magnitude of reduction in MPS in healthy individuals. This gap in knowledge is relevant because quantifying the magnitude of effect between different models of muscle disuse could offer valuable insights into future experimental designs and the development of targeted interventions to mitigate disuse‐induced muscle loss. Therefore, the aim of this systematic review and meta‐analysis was to synthesize data from studies investigating the effects of BR, ULLI and/or HDT on mixed MPS or MyoPS.

## 2 METHODS

This systematic review and meta‐analysis were conducted according to the Preferred Reporting Items for Systematic Reviews and Meta‐Analyses (PRISMA) guidelines (Page et al., 2021). The protocol was registered in the International Prospective Register of Systematic Reviews (PROSPERO) (CRD42024562343).

### 2.1 Search strategy

Two independent reviewers (K.P., P.T.M.) searched and extracted data from studies indexed in PubMed, Cochrane Library, Scopus, and Web of Science from inception until July 2025. The full search strategy and search terms used are described in Supporting information, Table S1. A manual search of references cited in the selected articles and published reviews was also performed. Searches were re‐run before submission to retrieve any additional studies that met our inclusion criteria.

### 2.2 Inclusion and exclusion criteria

Studies were included based on the following criteria: (i) randomized and non‐randomized controlled trials (RCTs) including adults above 18 years of age; (ii) healthy participants; and (iii) a study arm described as strict BR, ULLI, or 6° HDT. Published articles were excluded if they (i) were reviews or animal experiments; (ii) were not published as a full text; (iii) included participants younger than 18 years of age or with a chronic health condition; and (iv) included any study arm for which a pharmacological or non‐pharmacological intervention was introduced throughout the period of disuse.

### 2.3 Data extraction

Two authors independently extracted data (K.P., P.T.M.) which included first author name, date of publication, cohort and place where the study was undertaken, country of origin, sample size, sex, body mass index (BMI) and age of participants, muscle from which the biopsy was obtained, duration of muscle disuse model, pertinent details regarding tracer protocol employed during the metabolic trial, measurement of MPS (i.e., mixed or MyoPS) pre‐ and post‐ muscle disuse and whether dietary intake was controlled.

### 2.4 Risk of bias and quality of evidence

The risk of bias of the included studies was evaluated via the Risk of Bias 2 (RoB2) and the Risk of Bias for Non‐Randomized Studies of Interventions (ROBINS‐I) assessment tools (Sterne et al., 2016) by two authors (K.P., C.S.D.). RoB2 defines bias as ‘high’, ‘some concerns’ or ‘low’, while ROBINS‐I defines bias as ‘serious’, ‘moderate’, or ‘low’. Quality of evidence was assessed using the GRADE (Grading of Recommendations, Assessment, Development, and Evaluations) framework.

### 2.5 Statistical analysis

Quantitative data were treated as continuous measurements, and pre‐ to post‐muscle disuse changes in MPS were used to calculate mean differences. Where numerical data were not reported, graphical values from the figures were extracted using WebPlotDigitizer software. Statistical significance was assessed using the random effects model and inverse‐variance method, while a fixed effects model was employed when *P* for heterogeneity was >0.10. Any missing standard errors (SE) or standard deviations (SD) for pre‐ to post‐muscle disuse changes in MPS were estimated based on either 95% confidence intervals (95% CI) or SE/SD, or by calculating a correlation coefficient (Corr) from a known change from baseline SD derived from a similar study. In the absence of Corr values, conservative values of 0.7 and 0.5 were utilized based on the Cochrane Review (Deeks et al., 2019), for which a 0.7 constant was imputed into the main analyses.

Statistical heterogeneity between studies was assessed using the overlap of their 95% CI and expressed as measurements of Cochrane's *Q* (chi‐square test) and *I* ^2^. Data were classified as moderately heterogeneous with an *I* ^2^ value between 50% and 74.9%, and as highly heterogeneous with an *I* ^2^ value of ≥75% (Higgins et al., 2003).

The meta‐analysis was synthesized using R software, with a *P‐*value of <0.05 representing a significant effect of the muscle disuse model. Sensitivity analyses were conducted to assess the robustness of reported statistical results by excluding studies with an increased risk of bias in both models of muscle disuse. In addition, sensitivity analyses were performed regarding the effects of BR on mixed MPS in middle‐aged and older adults (mean age range: 52–67) and the effects of ULLI on MyoPS in young adults (mean age range: 20–23; one study only included an age range of 20–29 years), studies including males only, and studies that controlled for dietary intake. These analyses were informed by data availability. Studies that reported changes in fractional synthetic rate as ‘%/day’ were converted in‐text as ‘%/h’.

### 2.6 Publication bias

Publication bias was assessed by Egger's test and via visual inspection of funnel plots. In cases of publication bias, the trim‐and‐fill analysis was used to detect and adjust for publication bias (Duval & Tweedie, 2000). However, no publication bias was detected for studies that utilized BR or ULLI immobilization models of muscle disuse (*P* > 0.05). Details of *t*, *P*, *b* and 95% CI values alongside the presentation of funnel plots to study asymmetry are shown in the Supplementary File 1.

## 3 RESULTS

**FIGURE 1:**
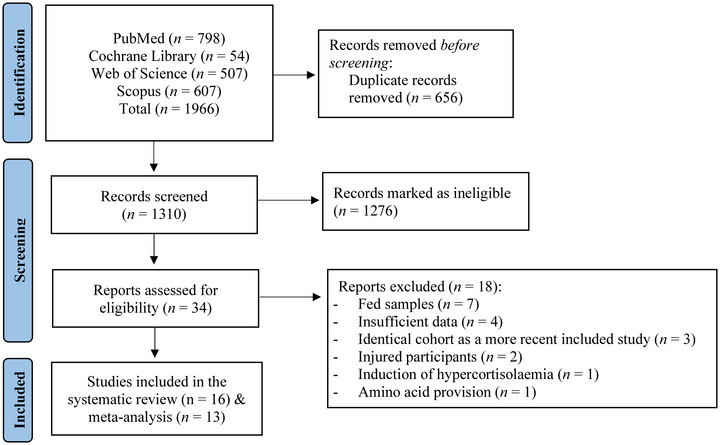
Flowchart of the search strategy.

The initial literature search yielded 1966 publications. After removing duplicates and non‐relevant abstracts, 34 full‐text articles were deemed eligible for inclusion. Of these articles, seven were excluded because MPS was calculated in the fed state only, four articles contained insufficient data, three consisted of an identical cohort as a more recent included study, two recruited injured participants, one article had induced hypercortisolaemia, and one had administered amino acids. Overall, 16 studies were included in the systematic review and 13 in the meta‐analysis (Figure 1). Four studies examined the impact of BR (Drummond et al., 2012; English et al., 2016; Kortebein et al., 2007; Tanner et al., 2015), nine the impact of unilateral lower limb immobilization (Brook et al., 2022; De Boer et al., 2007; Dideriksen et al., 2024; Edwards et al., 2020; Gamrin et al., 1998; Jameson et al., 2021; Kilroe et al., 2020; Mcglory et al., 2019; Mitchell et al., 2018) and three the impact of 6° HDT (Ferrando et al., 1997; Shur et al., 2024; Symons et al., 2009) on MPS rates. The studies that utilized the 6° HDT model were not included in the meta‐analysis due to the low sample size. Participant characteristics of included studies are displayed in Table 1.

**TABLE 1:** Study and participant characteristics of the included studies.

Study (year)	State	Total *n* (M/F)	Age	BMI	Infusion length	MPS assessment period	Study duration	Controlled dietary intake?
Drummond et al. (2012)	Bed rest	6 (5/1)	67.2 (1.7)	24.7 (0.9)	6 h	3 h	7 days	Yes
English et al. (2016)	Bed rest	9 (3/6)	52 (1)	24.7 (1.4)	8 h	3 h	14 days	Yes
Tanner et al. (2015) (Young)	Bed rest	14 (7/7)	22 (1)	23 (1)	7 h	2 h	5 days	Yes
Tanner et al. (2015) (Old)	Bed rest	9 (2/7)	66 (1)	25 (1)	7 h	2 h	5 days	Yes
Kortebein et al. (2007)	Bed rest	11	67 (1.4)	—	7 h	3 h	10 days	Yes
Brook et al. (2022)	Unilateral leg immobilization	9 (9/0)	22 (0.7)	24 (1)	7 h	4 days	4 days	Not reported
De Boer et al. (2007)	Unilateral leg immobilization	9 (9/0)	22 (1.3)	24 (1)	—	10–21 days	21 days	Not reported
Dideriksen et al. (2024)	Unilateral leg immobilization	18 (18/0)	60‐80	20‐30	5 h	2.5 h	14 days	No
Jameson et al. (2021)	Unilateral leg immobilization	11 (11/0)	20 (1)	23 (1)	10 days	7 days	7 days	Yes
Kilroe et al. (2020)	Unilateral leg immobilization	13 (13/0)	20 (1)	23.4 (0.9)	10 days	7 days	7 days	Yes
McGlory et al. (2019)	Unilateral leg immobilization	9 (0/9)	22 (1)	23.9 (0.8)	14+ days	14 days	14 days	Yes
Mitchell et al. (2018)	Unilateral leg immobilization	15 (15/0)	48.5 (0.6)	28.3 (0.8)	7 days	3 days	14 days	Yes
Edwards et al. (2020)	Unilateral leg immobilization	8 (8/0)	23	22.3 (0.9)	7 h	2.5 h	7 days	Yes
Gamrin et al. (1998)	Unilateral leg immobilization	5 (5/0)	Range: 20‐29	18.8‐24.3	—	—	10 days	Advised to maintain normal intake
Shur et al. (2024)	6° head‐down tilt	10 (10/0)	24 (1)	22.7 (0.6)	3 days	—	—	Yes
Symons et al. (2009)	6° head‐down tilt	7 (7/0)	27.3 (0.7)	26.5 (0.8)	21 days	—	30–60 min	Yes
Ferrando et al. (1997)	6° head‐down tilt	6 (6/0)	30 (2.4)	—	14 days	5 h	3 h	Yes

Data are expressed as mean (standard error) or range. BMI, body mass index.

### 3.1 Effects of bed rest on mixed MPS

**FIGURE 2:**
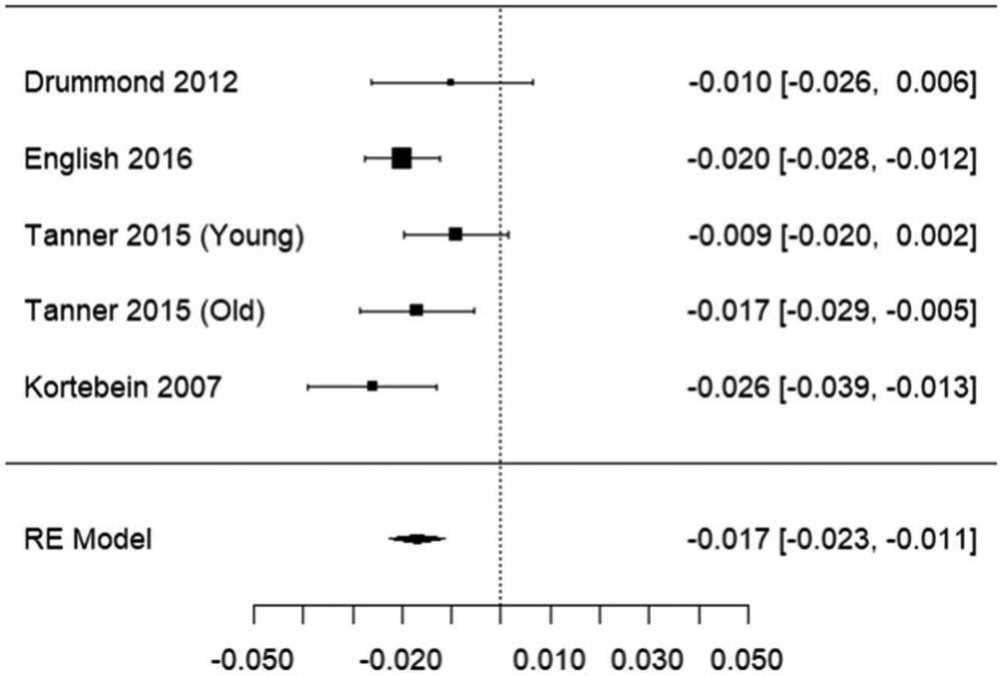
Effect of bed rest on mixed MPS.

The main analysis showed a significant reduction in mixed MPS after BR (*k* = 4; MD: −0.017%/h, 95% CI: −0.023 to −0.011, *I* ^2^ = 24%, *P* < 0.01, Figure 2). The sensitivity analysis, which excluded a study in young adults, did not modify the interpretation of the main findings (*k* = 4; MD: −0.019%/h, 95% CI: −0.025 to −0.014, *I* ^2^ = 4%, *P* < 0.01, Figure S1) or when Corr was set at 0.5 (*k* = 4; MD: −0.017%/h, 95% CI: −0.023 to −0.011, *I* ^2^ = 0%, *P* < 0.01, Figure S2).

### 3.2 Effects of unilateral lower limb immobilization on MyoPS

**FIGURE 3:**
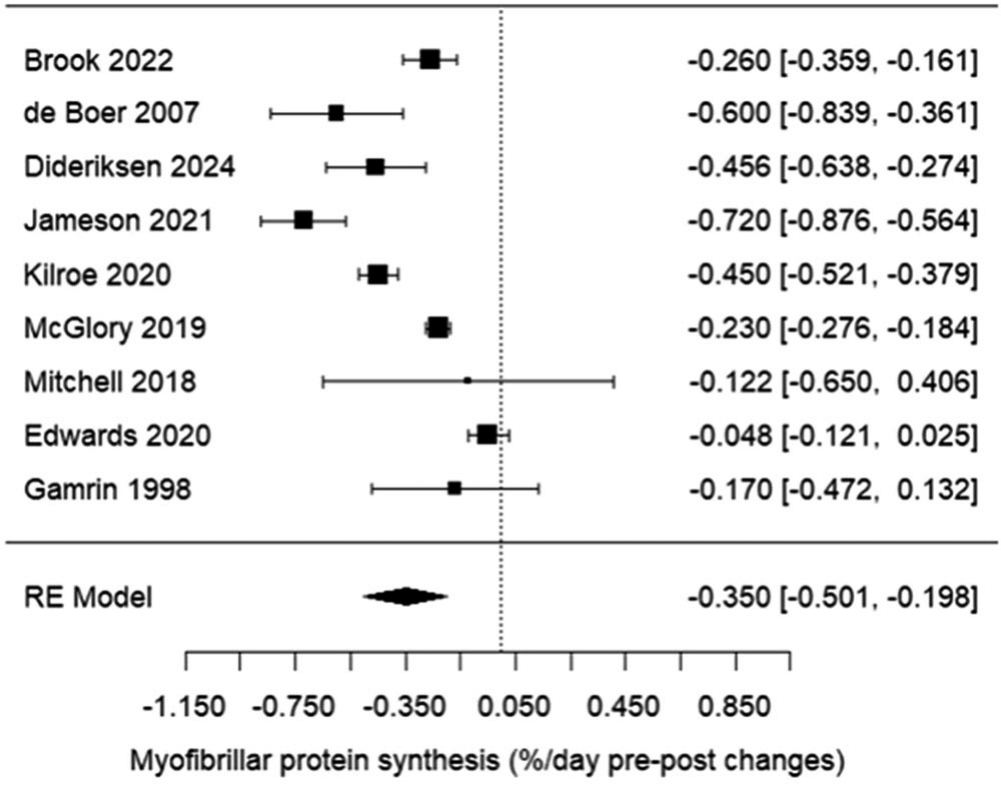
Effect of lower limb immobilization on MyoPS.

The main analysis showed a significant reduction in MyoPS following ULLI (*k* = 9; MD: −0.015%/h, 95% CI: −0.021 to −0.008, *I* ^2^ = 94%, *P* < 0.01, Figure 3). The sensitivity analysis, which excluded two studies in middle‐aged and older adults, showed no pre–post immobilization changes in MyoPS (*k* = 7; MD: −0.015%/h, 95% CI: −0.022 to −0.007, *I* ^2^ = 96%, *P* < 0.01, Figure S3). Similarly, no changes in MyoPS were observed following immobilization when males only were included in the statistical model (*k* = 8; MD: −0.015%/h, 95% CI: −0.022 to −0.008, *I* ^2^ = 92%, *P* < 0.01, Figure S4) or using a Corr value of 0.5 (*k* = 9; MD: −0.015%/h, 95% CI: −0.021 to −0.008, *I* ^2^ = 90%, *P* < 0.01, Figure S5). Furthermore, when only accounting for studies that controlled for diet, no statistical pre–post immobilization‐induced change in MyoPS was observed (*k* = 5; MD: −0.014%/h, 95% CI: −0.024 to −0.003, *I* ^2^ = 97%, *P* = 0.09, Figure S6). Our sensitivity analyses revealed identical results to the main analysis following exclusion of a study with a high risk of bias (*k* = 8; MD: −0.013%/h, 95% CI: −0.021 to −0.006, *I* ^2^ = 75%, *P* < 0.01, Figure S7).

### 3.3 Effects of 6° head‐down tilt on MPS

Three days of 6° HDT elicited a reduction in daily mixed MPS in young adults (*P* < 0.05) (Shur et al., 2024). In addition, a decline in mixed MPS was reported following a 3‐week period of HDT in young adults (MD (standard error) Baseline: 0.081 (0.0001)%/h; Post‐exposure: 0.042 (0.0001)%/h) (Symons et al., 2009) and similar results were observed for MyoPS after 2 weeks of HDT in young adults (MD (standard error) Baseline: 0.074 (0.011)%/h; Post‐exposure: 0.04 (0.007)%/h) (Ferrando et al., 1997).

### 3.4 Publication bias, risk of bias and quality of evidence

No publication bias was found in relation to mixed MPS and MyoPS, following BR and ULLI, respectively (Table S2). Regarding the risk of bias assessment, the majority of included studies did not blind or provide sufficient information regarding the blinding procedures, leading to a ‘high’ risk of bias score in this domain (Brook et al., 2022; De Boer et al., 2007; Gamrin et al., 1998; Jameson et al., 2021; Kilroe et al., 2020; Symons et al., 2009; Tanner et al., 2015). Additionally, multiple studies did not provide detailed information regarding their randomization process, thereby scoring a ‘moderate’ risk of bias in the first domain (Brook et al., 2022; De Boer et al., 2007; Edwards et al., 2020; English et al., 2016; Gamrin et al., 1998; Jameson et al., 2021; Kilroe et al., 2020; Symons et al., 2009; Tanner et al., 2015). The overall risk of bias of the randomized studies was considered ‘low’ and ‘some concerns’ for most studies, although two studies were scored as having a ‘high’ risk of bias (Kilroe et al., 2020; Symons et al., 2009) (Table S3). In relation to non‐randomized studies, two studies exhibited a ‘moderate’ risk due to missing data (Kortebein et al., 2007; Shur et al., 2024) and were deemed to pose a ‘serious’ risk of bias due to blinding challenges that could have affected the validity of primary outcome measurements (Kortebein et al., 2007; Shur et al., 2024). The overall risk of bias was considered ‘moderate’ in three out of four studies that utilized an HDT model of muscle disuse (Ferrando et al., 1997; Kortebein et al., 2007; Shur et al., 2024) (Table S4).

The evidence for mixed MPS under BR was rated as high certainty (Table S5). Despite a higher risk of bias, the lack of inconsistency, indirectness and imprecision, combined with a strong association, supported this rating, while the effect size indicated a modest reduction in mixed MPS (MD: −0.017%/h). Similarly, for MyoPS under ULLI, the evidence was also rated as high certainty. Despite some risk of bias being present, the absence of inconsistency, indirectness and imprecision and alongside a strong association, the large effect size also suggested a reduction in MyoPS (MD: −0.350%/day or −0.015%/h).

## 4 DISCUSSION

The aim of this systematic review and meta‐analysis was to synthesize data from studies investigating the effects of BR, ULLI and/or HDT on mixed MPS or MyoPS. By quantifying the magnitude of the effect of these models of muscle disuse on MPS, we provide valuable insights into developing suitable study designs aimed at targeted interventions to mitigate disuse‐induced muscle loss. This advance in understanding also has relevance in simulating scenarios that are more challenging to mimic within a research setting (e.g., space flight). Herein, we report significant reductions in MPS following muscle disuse irrespective of the disuse model employed (BR: MD: −0.017%/h; ULLI: MD: −0.015%/h) or the inclusion of sensitivity analyses (e.g., BR in middle‐to‐older adults; MD: −0.019%/h; ULLI in males only: MD: −0.015%/h; ULLI in younger adults: MD: −0.015%/h; ULLI in studies controlling for diet: MD: −0.014%/h). Whilst a meta‐analysis was not conducted on studies that utilized the HDT model due to the limited availability and heterogeneous nature of the data, we observed a similar magnitude of decline in MPS with HDT.

### 4.1 Comparing models of muscle disuse

Our observation of a similar reduction in MPS across muscle disuse models (i.e., BR, ULLI, HDT) is indicative of a comparable magnitude of disuse‐induced muscle atrophy (Deane et al., 2024). Whilst limited data are available regarding HDT, disuse‐induced changes in MPS were similar for BR and ULLI (−0.017%/h vs. −0.015%/h, respectively; *P* < 0.05). These data indicate that the magnitude of impairment in MPS is comparable between muscle disuse models, and thus, researchers should prioritize selecting the disuse model that most closely aligns with the participant characteristics during the conception of future study designs. Indeed, whilst BR and ULLI are commonly utilized as models to study physiological perturbations associated with muscle disuse atrophy, each model has its own advantages and limitations within a research setting. For example, BR simulates a model of whole‐body disuse most relevant to prolonged inactivity or microgravity, and for studying the systemic effects of disuse. BR is also relevant to clinical populations during prolonged hospitalization and those following a sedentary lifestyle, whilst affording a controlled environment whereby researchers standardize extraneous variables such as dietary intake and physical activity, which are known to influence MPS (Atherton & Smith, 2012). Conversely, BR presents ethical and logistical challenges and is often labour‐intensive and not financially viable to conduct. Similarly, BR does not provide insight into muscle‐specific effects of disuse, and prolonged inactivity may also influence various psychological and behavioural factors, thus presenting the possibility of additional confounding factors. Another consideration relates to temporal changes in MPS during prolonged periods of disuse that, from a quantitative perspective, likely impact the direct translation of shorter periods of experimental disuse to prolonged disuse settings (English et al., 2016).

ULLI, whereby a single limb is immobilized, offers key advantages as a model of muscle disuse, including the ability to isolate localized, muscle‐specific effects and use of the contralateral limb as an internal control, thereby minimizing inter‐subject variability (Pavis et al., 2023; Phillips & McGlory, 2014). Conversely, ULLI is not representative of whole‐body disuse, which is often accompanied by significant metabolic and cardiovascular implications. Therefore, ULLI may not represent and/or replicate the systemic demands of whole‐body bed rest (e.g., bedridden individuals, astronauts) or the clinical manifestation of muscle loss. According to our meta‐analysis, studies that controlled for dietary intake demonstrated no significant pre‐ to post changes in MPS in response to ULLI. These findings suggest that dietary adherence to adequate energy and protein intake may be prudent factors in mitigating the decline in muscle mass associated with ULLI. Conversely, when dietary protein intake was controlled at sub‐optimal levels (1.0 g/kg body mass (BM)/day over 7 days), Edwards et al. (2020) observed an impaired postprandial response of MyoPS to 20 g of ingested milk protein in the immobilized lower limb versus control in young adults (Edwards et al., 2020). Likewise, the ingestion of 20 g of dairy protein (leading to a dietary protein intake of 1.08 g/kg BM/day) versus placebo (leading to a dietary protein intake of 0.79 g/kg BM/day) did not attenuate losses in muscle mass and function after 14 days of ULLI (Mitchell et al., 2018). In contrast, while a controlled diet is often provided during BR, muscle atrophy is still prevalent. This decline in muscle mass may be primarily attributed to inadequate protein intake, given that dietary protein intake is typically aligned with the RDA of ∼0.8 g/kg BM/day in clinical scenarios. This observation is particularly concerning during hospitalization, especially in older adults at risk of malnutrition, who, when calculating wastage, often consume a protein intake of <0.65 g/kg/day despite being provided the protein RDA (Weijzen et al., 2020). Taken together, these data highlight the greater potential for dietary strategies to ameliorate disuse‐induced muscle atrophy caused by ULLI compared with BR when dietary protein is provided at the RDA.

Interestingly, the utility of ULLI as an experimental model of disuse may primarily be dependent on the experimental design and subject compliance. For example, alterations in joint position and the type of immobilization (e.g., casting, bracing) represent both a strength and a limitation of ULLI by providing versatility but also represent a confounding factor that contributes to the heterogeneity of responses to disuse and the rate of muscle atrophy. It is also possible that compensatory adaptations as a result of ULLI may occur in the non‐immobilized control limb (MacInnis et al., 2017; Phillips & McGlory, 2014; Tesch et al., 2016). While this rationale is plausible, to our knowledge, no direct experimental evidence currently exists. Nonetheless, this idea highlights the importance of practical considerations such as the use of continuous temperature monitoring to monitor muscle activity in the immobilized limb to ensure participant compliance, and the need to account for travel to the laboratory when using ULLI as a model of experimental disuse.

### 4.2 Sensitivity and sub‐group analysis

As part of our meta‐analysis, we conducted a sensitivity analysis that revealed age‐specific effects on MPS rates when both BR and ULLI models of disuse were pooled. For instance, the reduction in MPS during BR was marginally greater in middle‐aged to older adults (MD: –0.019%/h) compared to the overall analysis, which included young adults and revealed a modestly reduced numerical decline in MPS (MD: –0.017%/h). Given that we observed no statistical differences in MPS between models of disuse, this sub‐group analysis suggests that older adults are more susceptible to the effects of disuse on MPS and/or that older adults may be more susceptible to the whole‐body effects of BR on MPS. These observations may be partially related to the systemic nature of disuse under BR conditions compared to ULLI (i.e., localized muscle disuse). For instance, the contralateral active limb, as well as the upper body, can remain more active during ULLI compared to BR, potentially alleviating a degree of negative systemic effects on MPS (Preobrazenski et al., 2023; Wall et al., 2016). Accordingly, a compensatory increase in physical activity may be observed in the non‐immobilized limb, thus partially mitigating the disuse‐induced decline in MPS. Our finding of a more pronounced effect of BR on impaired MPS rates in middle‐to‐older aged adults (i.e., MD: −0.019%/h) may also be explained, at least in part, by the presence of anabolic resistance, in which a greater stimulus of protein intake and/or mechanical loading may be required to stimulate MPS with advancing age (Cuthbertson et al., 2005; Kumar et al., 2009), albeit AR in the context of disuse is understudied. Nevertheless, our data suggest that the short‐term effects of ULLI on MPS may be influenced by confounding variables pertaining to the control of dietary intake, given that no statistically significant differences were observed when data were pooled from studies that strictly controlled diet within a laboratory setting. Moving forward, future studies that control dietary intake are required to consolidate our age‐related subgroup analysis, given that age may modulate the response of MPS during disuse. Taken together, these confounding factors should be considered in the context of study design when investigating the physiological impact of muscle disuse and interventions to counteract disuse‐induced perturbations in muscle protein turnover.

### 4.3 Strengths and limitations

The inclusion of BR, ULLI and HDT disuse models within our systematic review and meta‐analysis enabled a comprehensive understanding of how different models may impact mixed MPS and MyoPS. In addition, we employed multiple sensitivity analyses using studies conducted in males only or those that accounted for dietary intake, in order to increase the robustness of findings. However, our analyses were prone to several limitations. First, although pre–post changes in MPS may be predominantly attributed to muscle disuse, the absence of a control group limits the ability to account for external variables that may contribute to changes in MPS. The use of baseline data from pre‐muscle disuse MPS values provides a reasonable comparator to infer disuse‐specific effects, particularly when studies controlled for known confounders. In addition, the majority of eligible studies were conducted in healthy young adults, thereby limiting translation of findings to healthy middle‐aged or older adults, or patient groups subject to disuse conditions (i.e., critical illness, surgery). Moreover, the majority of studies were conducted in males only (11/16 studies; 69%), four studies included both males and females (4/16, 25%), while only one study included females only (1/16, 6%), thus highlighting the current under‐representation of females within the field of muscle disuse physiology that requires attention. Another limitation is the inconsistency in the assessment of mixed MPS and MyoPS across included studies. Typically, MyoPS was not evaluated in bed rest studies, while mixed MPS was not assessed in unilateral limb immobilization studies, which limits the comparability of our findings. Finally, multiple studies reported an increased risk of bias due to lack of dietary intake control, deviations from intended interventions, and selective reporting, which may affect the reliability of data presented and thus warrant consideration during the design of future experimental studies on muscle disuse.

### 4.4 Conclusion

This systematic review and meta‐analysis demonstrated that muscle disuse, regardless of the disuse model implemented, leads to significant reductions in mixed MPS and MyoPS in healthy young, middle‐aged and older adults. The magnitude of the effect of BR and ULLI on MPS was comparable between models, with important implications for future studies designed to examine the efficacy of targeted interventions to mitigate disuse‐induced muscle loss. Whilst our data suggest minimal differences between BR and ULLI models in terms of impaired MPS rates, both models are valuable for advancing understanding of the impact of disuse on muscle protein turnover. Accordingly, the choice of the disuse model depends on many factors, including the research question, ethical considerations and logistical constraints. With a focus on mimicking clinically relevant scenarios (e.g., intensive care unit patients, astronauts), BR more closely mimics real‐world conditions, whilst ULLI offers a short‐term, cost‐effective practical alternative to study physiological mechanisms associated with disuse‐induced muscle atrophy and targeted therapeutic interventions.

## AUTHOR CONTRIBUTIONS

*Conceptualizing the idea of this project*: Konstantinos Prokopidis, Paul T. Morgan and David D. Church. *Extracting data from the included studies*: Konstantinos Prokopidis and Paul T. Morgan. *Writing the manuscript*: Konstantinos Prokopidis and Paul T. Morgan. *Conducting the analyses*: Konstantinos Prokopidis. *Risk of bias assessment performed by*: Konstantinos Prokopidis and Colleen S. Deane. Colleen S. Deane, Paul T. Morgan, Oliver C. Witard. *Revising the manuscript*: David D. Church. All authors have read and approved the final version of this manuscript and agree to be accountable for all aspects of the work in ensuring that questions related to the accuracy or integrity of any part of the work are appropriately investigated and resolved. All persons designated as authors qualify for authorship, and all those who qualify for authorship are listed.

## CONFLICT OF INTEREST

D.C.C has received research funding from the National Pork Board, Beef Checkoff Program managed by the NCBA, National Dairy Council; has performed freelance work for Soy Connection funded by U.S. Soy; is on the advisory board for Shifted Supplements; and has received travel reimbursement from Protein PACT. There are no relationships or activities that could appear to have influenced the submitted work. The other authors report no conflicts of interest relevant to the publication of this manuscript.

## FUNDING INFORMATION

This work received no external funding.

## Supporting information




**Figure S1**. Effect of bed rest on mixed MPS in middle‐to‐older adults.

**Figure S2**. Effect of bed rest on mixed MPS using a Corr value of 0.5.

**Figure S3**. Effect of lower limb immobilization on MyoPS in younger adults.

**Figure S4**. Effect of lower limb immobilization on MyoPS in males only.

**Figure S5**. Effect of lower limb immobilization using a Corr value of 0.5.

**Figure S6**. Effect of lower limb immobilization on MyoPS using studies that controlled for diet.

**Figure S7**. Effect of lower limb immobilization on MyoPS on studies without a high risk of bias.

Supporting Information

**Table S1. **Search terms employed in the screening based on title, abstract, and keywords in the literature search.

**Table S2**. Publication bias using Egger's test.

**Table S3**. Risk of bias assessment of the included studies using the RoB2 tool.

**Table S4**. Risk of bias assessment of the included studies using the ROBINS‐I tool.

**Table S5**. Quality of evidence based on GRADE assessment.

## Data Availability

Data is available upon request.
