# Supplementary figures and images for: The effect of bed rest, unilateral limb immobilization and head‐down tilt on muscle protein synthesis: A systematic review and meta‐analysis
Source: Exp Physiol. 2025 Oct 30:10.1113/EP092474. Online ahead of print. doi: 10.1113/EP092474 (PMC13394532; doi:10.1113/EP092474)

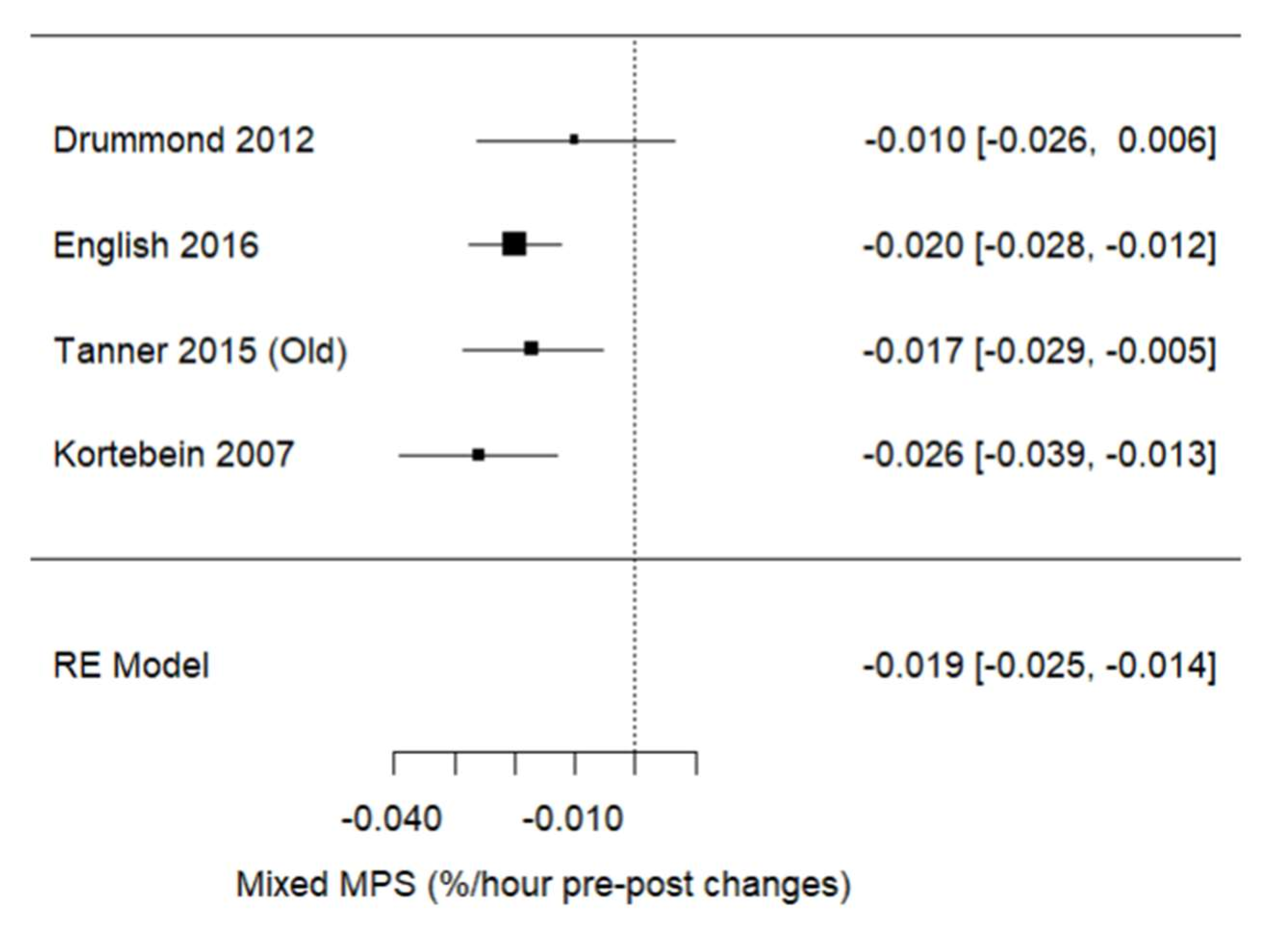

Supplement: Supplementary file 1 — Figure S1. Effect of bed rest on mixed MPS in middle‐to‐older adults. [file EPH-9999-0-s007.tif]

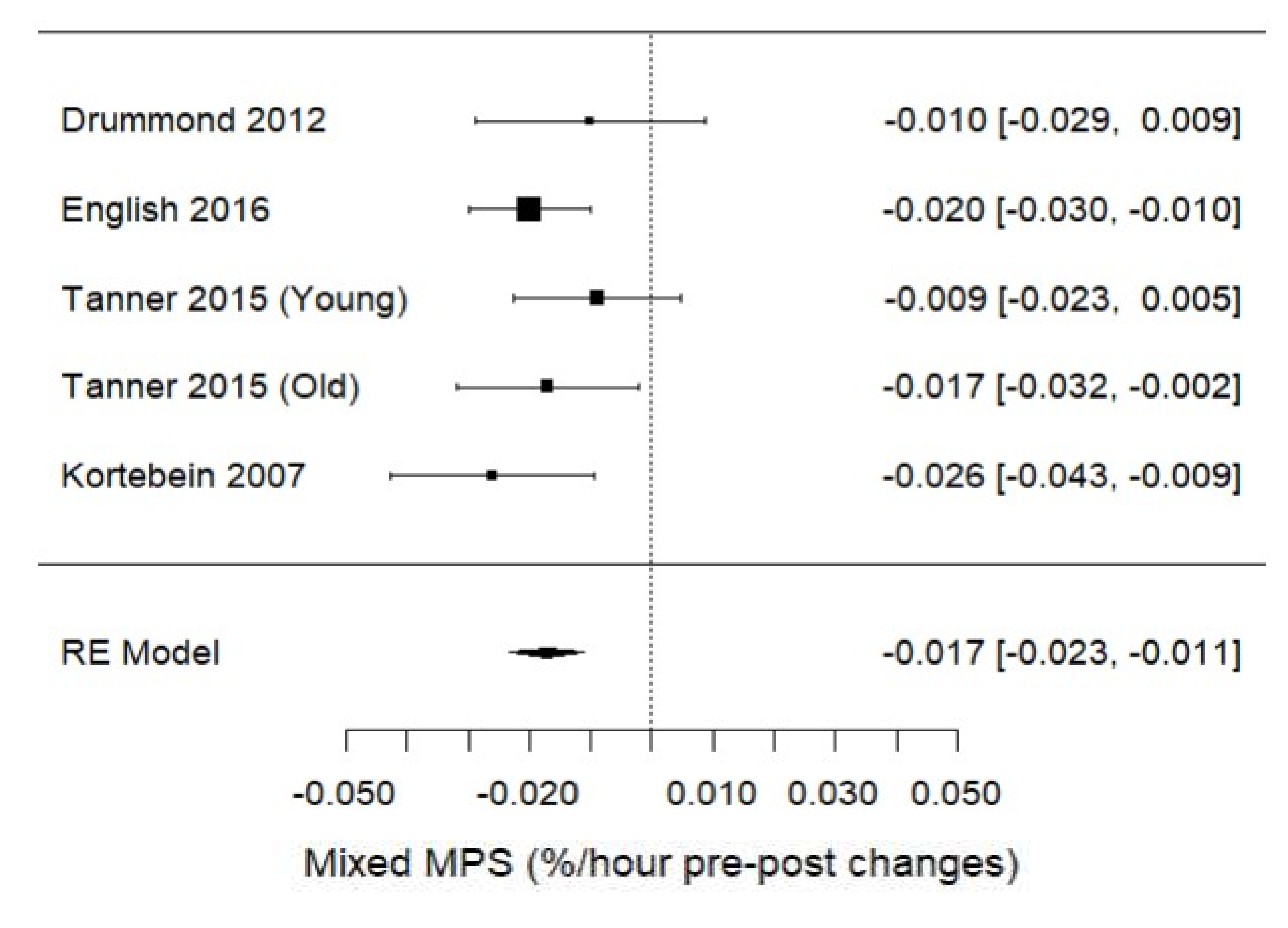

Supplement: Supplementary file 2 — Figure S2. Effect of bed rest on mixed MPS using a Corr value of 0.5. [file EPH-9999-0-s012.tif]

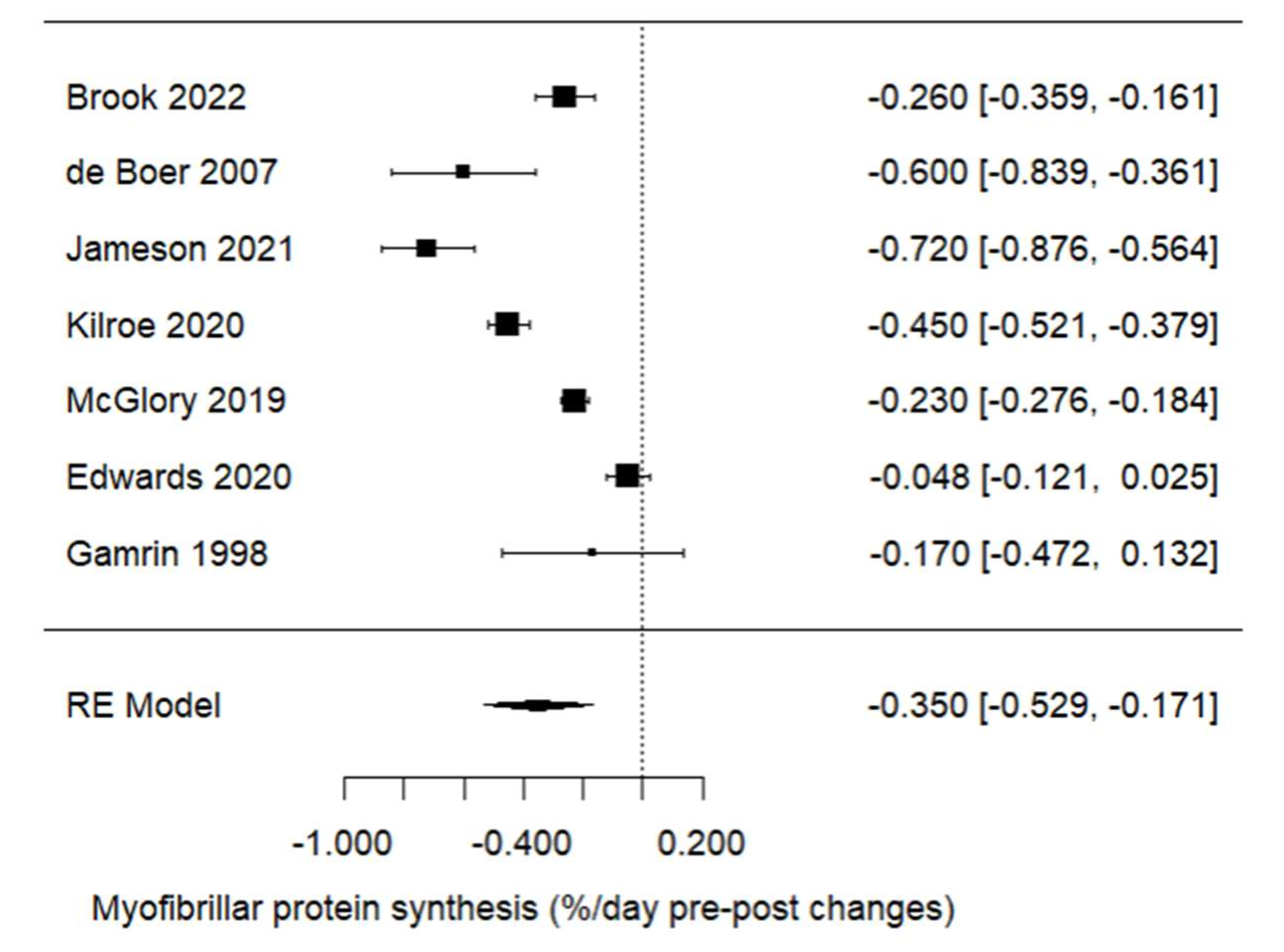

Supplement: Supplementary file 3 — Figure S3. Effect of lower limb immobilization on MyoPS in younger adults. [file EPH-9999-0-s009.tif]

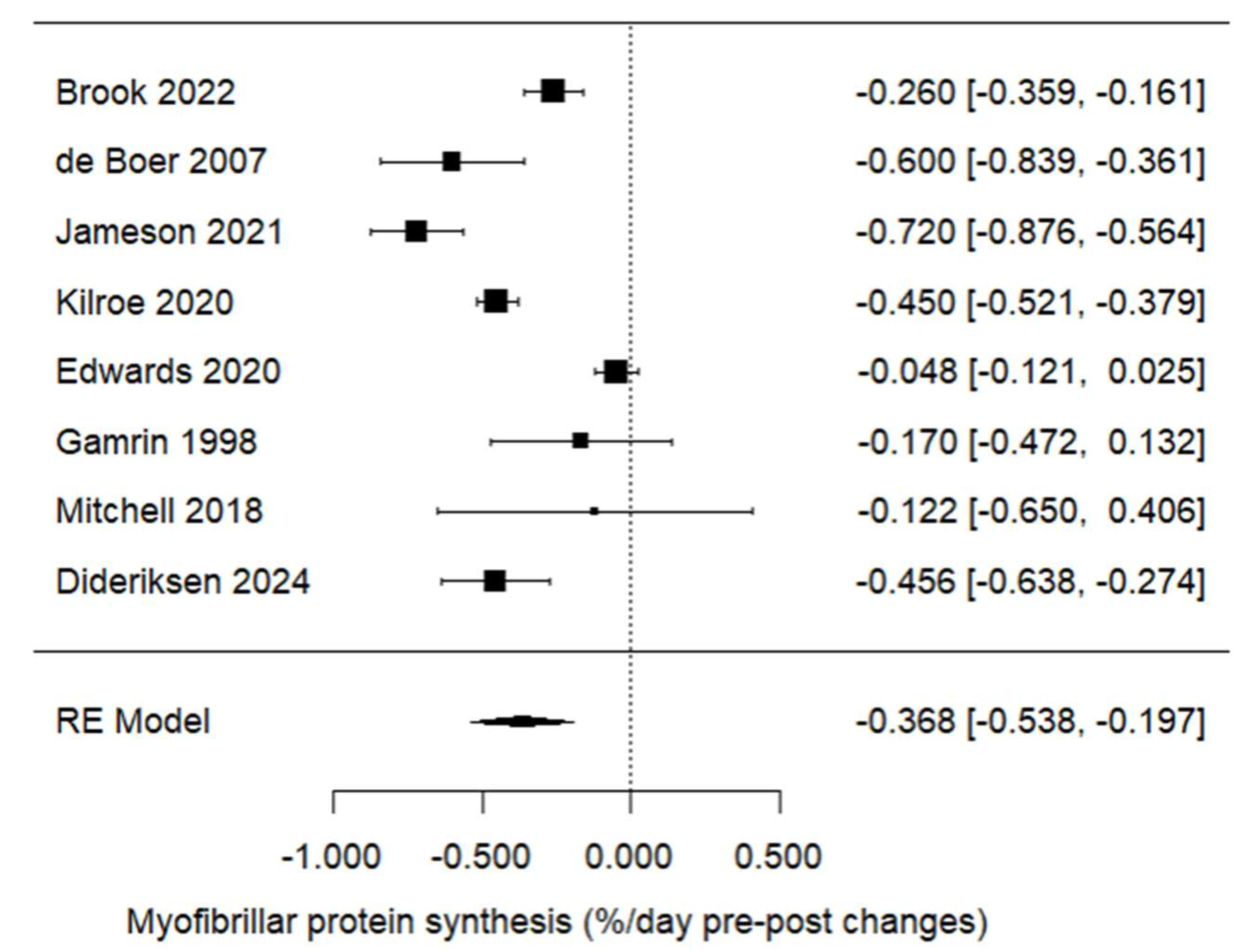

Supplement: Supplementary file 4 — Figure S4. Effect of lower limb immobilization on MyoPS in males only. [file EPH-9999-0-s013.tif]

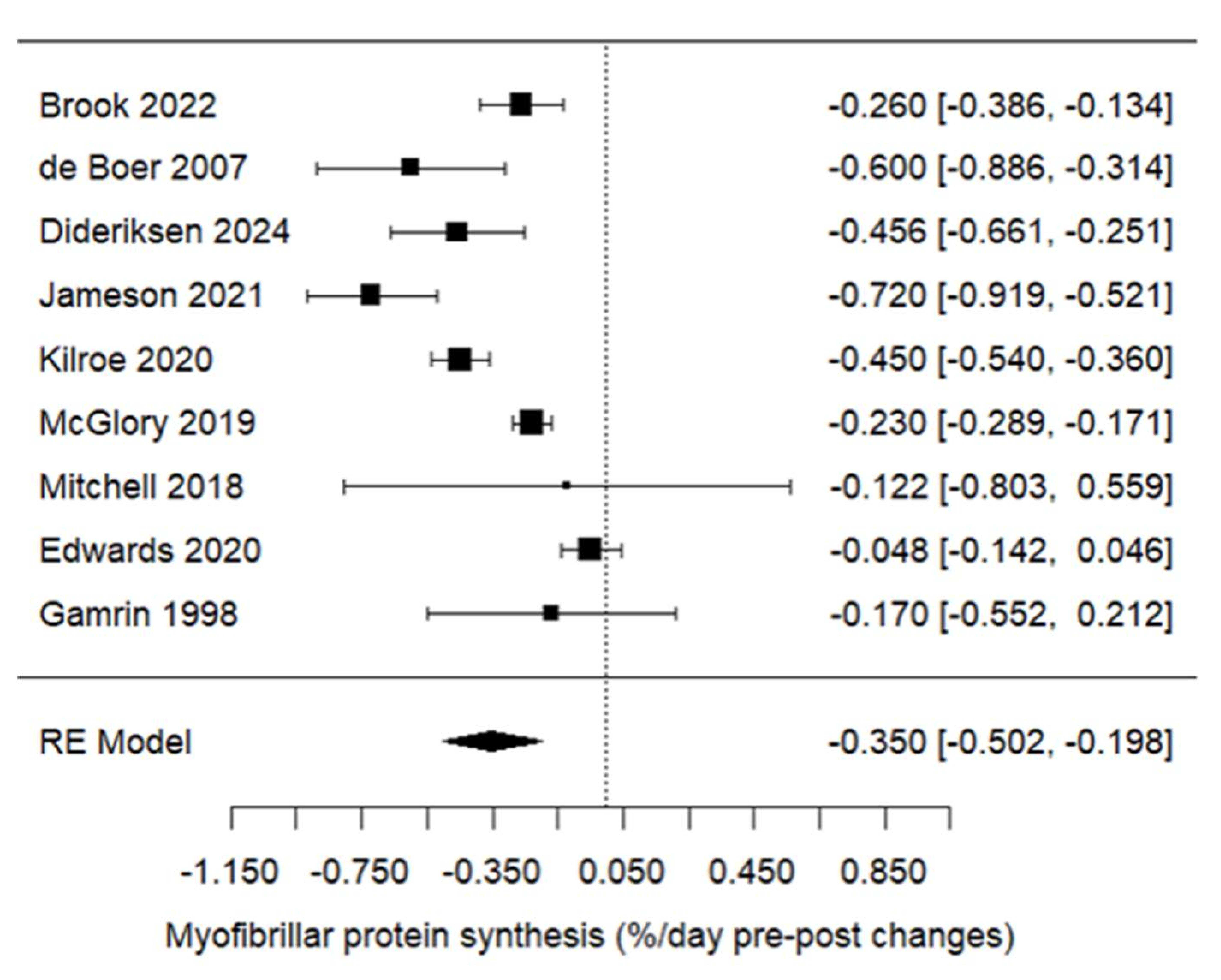

Supplement: Supplementary file 5 — Figure S5. Effect of lower limb immobilization using a Corr value of 0.5. [file EPH-9999-0-s005.tif]

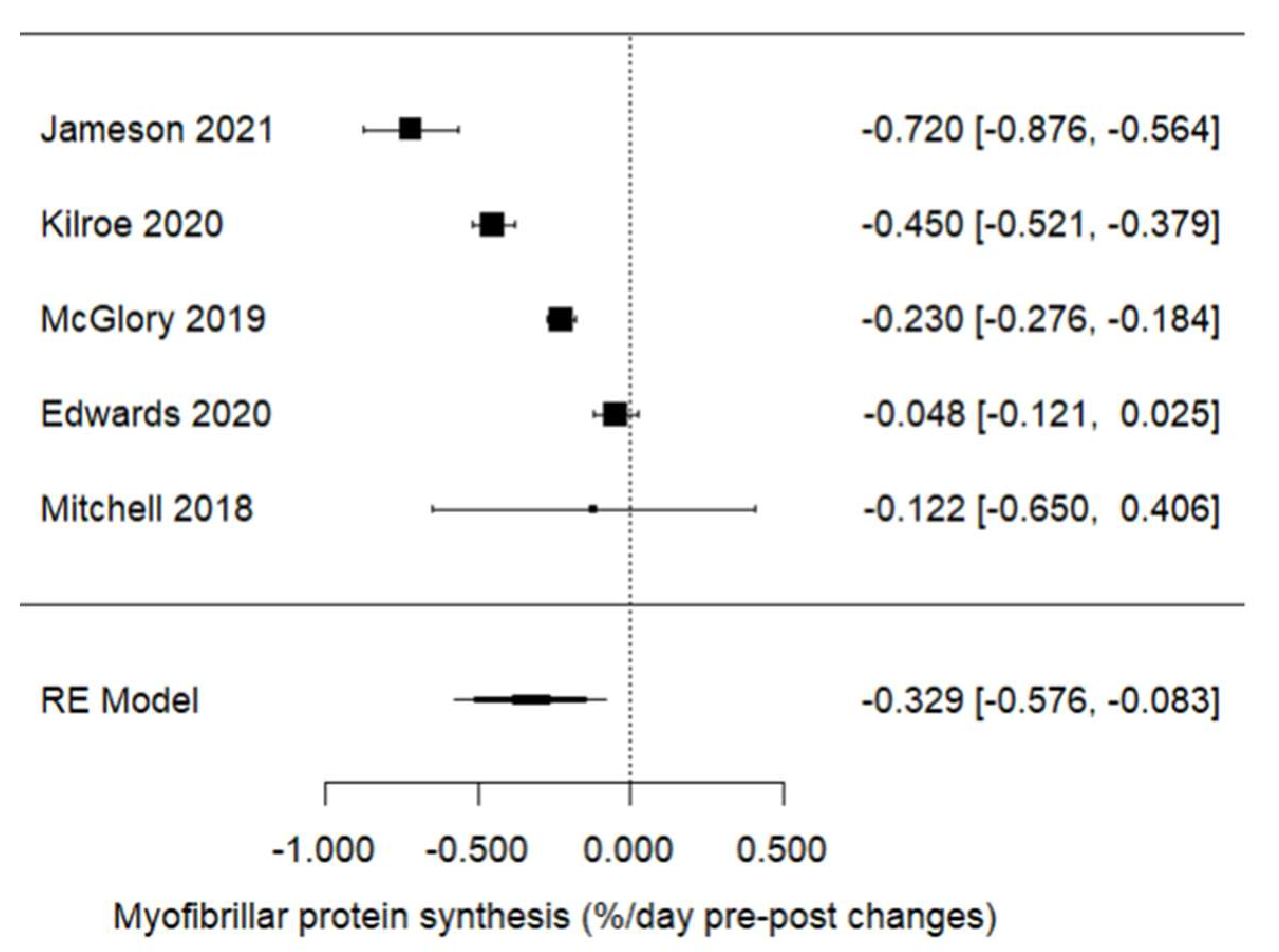

Supplement: Supplementary file 6 — Figure S6. Effect of lower limb immobilization on MyoPS using studies that controlled for diet. [file EPH-9999-0-s003.tif]

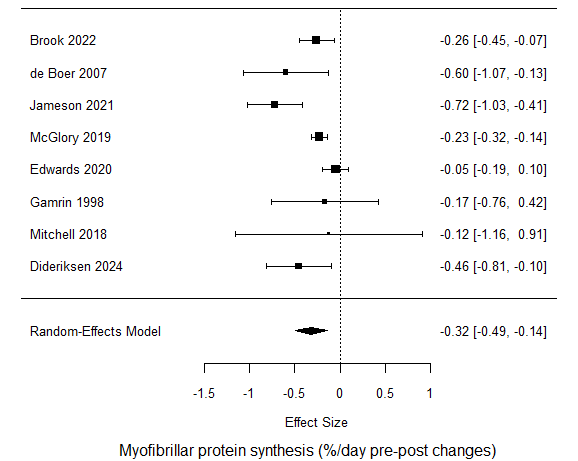

Supplement: Supplementary file 7 — Figure S7. Effect of lower limb immobilization on MyoPS on studies without a high risk of bias. [file EPH-9999-0-s011.tif]

**Table S3.** Risk of bias assessment of the included studies using the RoB2 tool.


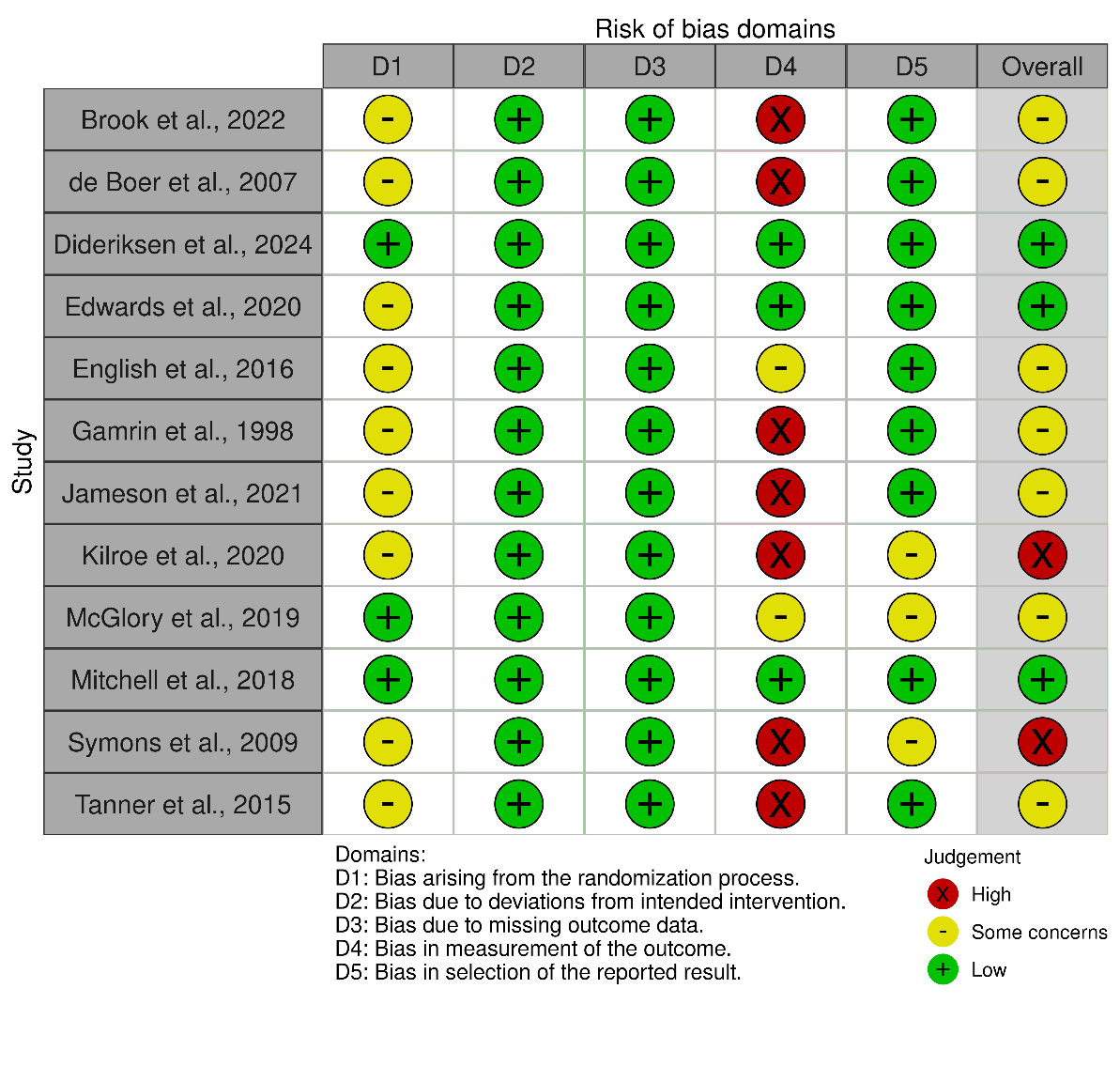

Supplement: Supplementary file 11 — Table S3. Risk of bias assessment of the included studies using the RoB2 tool. [file EPH-9999-0-s002.docx]

**Table S4.** Risk of bias assessment of the included studies using the ROBINS-I tool.


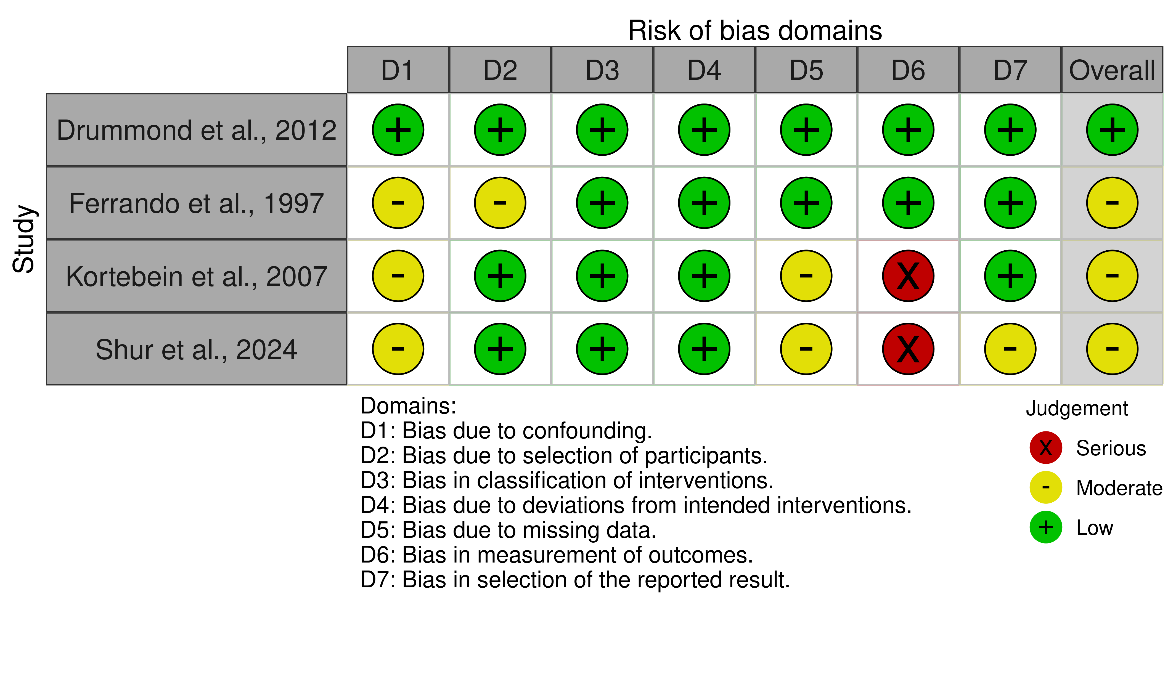

Supplement: Supplementary file 12 — Table S4. Risk of bias assessment of the included studies using the ROBINS‐I tool. [file EPH-9999-0-s004.docx]
