# Supplementary material for: The effect of bed rest, unilateral limb immobilization and head‐down tilt on muscle protein synthesis: A systematic review and meta‐analysis
Source: Exp Physiol. 2025 Oct 30:10.1113/EP092474. Online ahead of print. doi: 10.1113/EP092474 (PMC13394532; doi:10.1113/EP092474)

**Bed Rest – Mixed MPS – Egger’s Test**


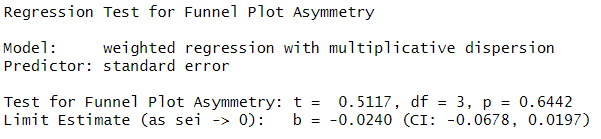


**Funnel Plot - Bed Rest – Mixed MPS**


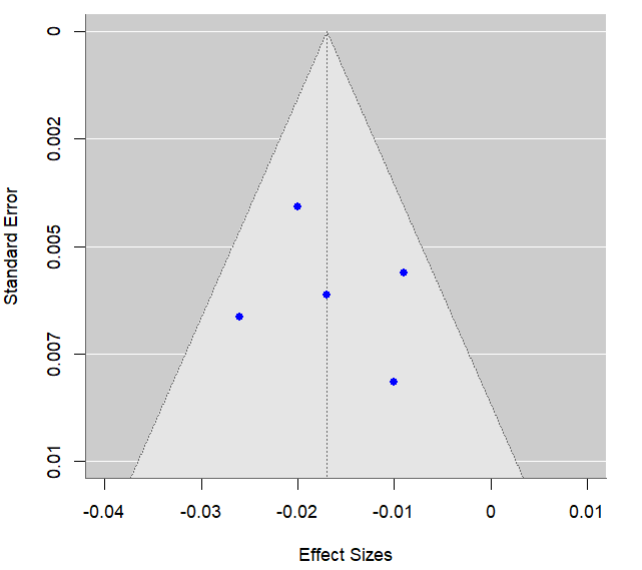


**Immobilization – myoMPS – Egger’s Test**

**
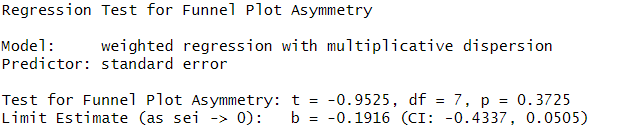
**

**Funnel Plot - Immobilization – myoMPS**


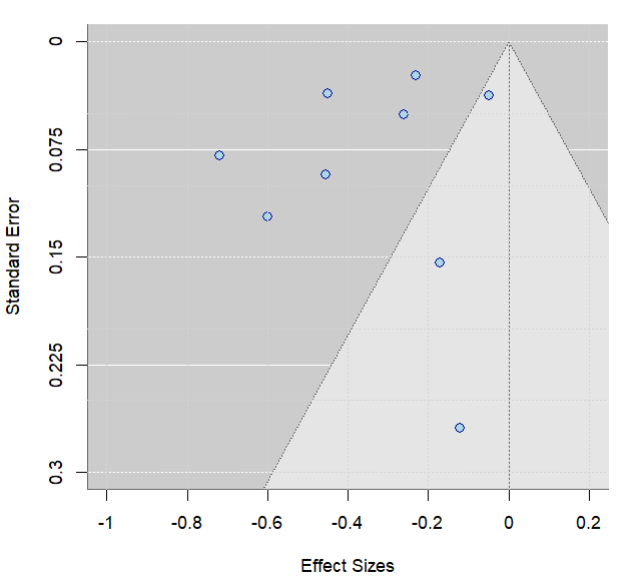

Supplement: Supplementary file 8 — Supporting Information [file EPH-9999-0-s008.docx]
