# Supplementary material for: The effect of bed rest, unilateral limb immobilization and head‐down tilt on muscle protein synthesis: A systematic review and meta‐analysis
Source: Exp Physiol. 2025 Oct 30:10.1113/EP092474. Online ahead of print. doi: 10.1113/EP092474 (PMC13394532; doi:10.1113/EP092474)
Supplement: Supplementary file 9 — Table S1. Search terms employed in the screening based on title, abstract, and keywords in the literature search. [file EPH-9999-0-s010.docx]

**Table S1.**Search terms employed in the screening based on title, abstract, and keywords in the literature search.

| **Database** | **Search terms** |
| --- | --- |
|  |  |
| PubMed | ("hypokinesia" OR "hypoactivity" OR "muscle disuse" OR "disuse atrophy" OR "immobiliz*" OR "immobilis*" OR "bed rest"  OR "bedridden" OR "bedrest" OR "head-down tilt" OR "microgravity" OR "weightlessness") AND ("myofibrillar protein synthesis"  OR "muscle protein synth*" OR "fractional synthetic rate*" OR "protein turnover" OR "protein metabolism" OR  "muscle protein fractional*" OR "protein kinetics") |
| Cochrane Library | ("hypokinesia" OR "hypoactivity" OR "muscle disuse" OR "disuse atrophy" OR "immobiliz*" OR "immobilis*" OR "bed rest"  OR "bedridden" OR "bedrest" OR "head-down tilt" OR "microgravity" OR "weightlessness") AND ("myofibrillar protein synthesis"  OR "muscle protein synth*" OR "fractional synthetic rate*" OR "protein turnover" OR "protein metabolism" OR  "muscle protein fractional*" OR "protein kinetics") |
| Web of Science | ("hypokinesia" OR "hypoactivity" OR "muscle disuse" OR "disuse atrophy" OR "immobiliz*" OR "immobilis*" OR "bed rest"  OR "bedridden" OR "bedrest" OR "head-down tilt" OR "microgravity" OR "weightlessness") AND ("myofibrillar protein synthesis"  OR "muscle protein synth*" OR "fractional synthetic rate*" OR "protein turnover" OR "protein metabolism" OR  "muscle protein fractional*" OR "protein kinetics") |
| Scopus | ("hypokinesia" OR "hypoactivity" OR "muscle disuse" OR "disuse atrophy" OR "immobiliz*" OR "immobilis*" OR "bed rest"  OR "bedridden" OR "bedrest" OR "head-down tilt" OR "microgravity" OR "weightlessness") AND ("myofibrillar protein synthesis"  OR "muscle protein synth*" OR "fractional synthetic rate*" OR "protein turnover" OR "protein metabolism" OR  "muscle protein fractional*" OR "protein kinetics") |
