# Supplementary material for: The effect of bed rest, unilateral limb immobilization and head‐down tilt on muscle protein synthesis: A systematic review and meta‐analysis
Source: Exp Physiol. 2025 Oct 30:10.1113/EP092474. Online ahead of print. doi: 10.1113/EP092474 (PMC13394532; doi:10.1113/EP092474)
Supplement: Supplementary file 10 — Table S2. Publication bias using Egger's test. [file EPH-9999-0-s006.docx]

**Table S2.** Publication bias using Egger’s test.

|  | ***p*** | ***t*** | ***b*** | **95%CI** |
| --- | --- | --- | --- | --- |
| Bed Rest – mixed MPS | 0.64 | 0.5117 | -0.024 | -0.0678 – 0.0197 |
| Immobilization – MyoPS | 0.37 | -0.9525 | -0.1916 | -0.4337 – 0.0505 |
