# Supplementary material for: The effect of bed rest, unilateral limb immobilization and head‐down tilt on muscle protein synthesis: A systematic review and meta‐analysis
Source: Exp Physiol. 2025 Oct 30:10.1113/EP092474. Online ahead of print. doi: 10.1113/EP092474 (PMC13394532; doi:10.1113/EP092474)
Supplement: Supplementary file 13 — Table S5. Quality of evidence based on GRADE assessment. [file EPH-9999-0-s001.docx]

**Table S5.** Quality of evidence based on GRADE assessment.

**Bed Rest and mixed MPS.**

| **Certainty assessment** | | | | | | | | **Effect** | **Certainty** | **Importance** |
| --- | --- | --- | --- | --- | --- | --- | --- | --- | --- | --- |
| **№ of studies** | **Study design** | **Risk of bias** | **Inconsistency** | **Indirectness** | **Imprecision** | **Other considerations** | **№ of patients** | **Absolute (95% CI)** |  |  |
| **Mixed muscle protein synthesis (Scale from: -0.1 to 0.1)** | | | | | | | | | | |
| 4 | randomised trials | serious | not serious | not serious | not serious | strong association | 49 | MD **0.017 lower** (0.023 lower to 0.011 lower) per hour | ⨁⨁⨁⨁ High | Important |

**Unilateral lower limb immobilization compared and myofibrillar protein synthesis.**

| **Certainty assessment** | | | | | | | | **Effect** | **Certainty** | **Importance** |
| --- | --- | --- | --- | --- | --- | --- | --- | --- | --- | --- |
| **№ of studies** | **Study design** | **Risk of bias** | **Inconsistency** | **Indirectness** | **Imprecision** | **Other considerations** | **№ of patients** | **Absolute (95% CI)** |  |  |
| **Myofibrillar protein synthesis (Scale from: -0.4 to 0.1)** | | | | | | | | | | |
| 9 | randomised trials | serious | not serious | not serious | not serious | strong association | 97 | MD **0.350 lower** (0.501 lower to 0.198 lower) per day | ⨁⨁⨁⨁ High | Critical |
